# Quality-Controlled Generative Augmentation for North Atlantic Right Whale Upcall Detection Using Contour 1D-VAE

**DOI:** 10.3390/s26154679

**Published:** 2026-07-23

**Authors:** Jongmin Ahn, Geun-Ho Park, Ho-Seuk Bae, Donghun Lee

**Affiliations:** Agency for Defense Development, Changwon-si 516852, Republic of Korea

**Keywords:** North Atlantic right whale, variational autoencoder, data augmentation, diffusion model, acoustic sensing

## Abstract

This study proposes a Variational AutoEncoder (VAE)-based Quality Controlled (QC) generative augmentation framework for North Atlantic right whale (NARW) upcall detection. Existing generative augmentation methods can generate synthetic samples; however, they do not provide a sample-level criterion for determining whether each generated sample is a positive sample that contributes to improved detector performance or a synthetic outlier that should be removed. This study learns manually extracted upcall frequency contours using a 1D-VAE and evaluates generated contour candidates in a 10-dimensional acoustic morphology feature space. The QC score is computed with respect to the reference distribution of manually extracted real upcall contours, and stochastic acceptance probabilities for borderline samples around the hard threshold are calibrated using same-call manual re-extraction variability. QC-passed contours are converted into detector training spectrograms using smooth amplitude modulation and Gaussian noise injection based on SNR statistics. Using 30,000 acoustic segments from the Kaggle NARW dataset, Original, Denoising Diffusion Probabilistic Models (DDPM), Contour-VAE without QC, and VAE-QC conditions were compared under the same detector and augmentation budget. VAE-QC with α = 0.97 achieved the highest mean AUC of 0.901, outperforming Original training (0.802), DDPM (0.845), and Contour-VAE without QC (0.810). Feature distribution and QC-score analyses further showed that VAE-QC suppresses morphology outlier tails observed in unfiltered generation. These results indicate that the key factor in generative augmentation is the QC process that defines feature boundaries useful for detector learning and selects synthetic positive samples accordingly.

## 1. Introduction

Underwater acoustic sensing has become a core technology for long-term monitoring of marine environments and marine organisms. In particular, passive acoustic monitoring (PAM) can continuously acquire long-duration data through hydrophones or acoustic sensor arrays deployed underwater, making it useful for estimating the presence of marine mammals that are difficult to observe visually or occur infrequently. In PAM-based observations, automatic detection of target bioacoustic events from large volumes of sensor-acquired acoustic data is essential, and signal-processing and deep learning-based detectors have recently been applied to various marine mammal vocalization detection tasks [1,2,3,4,5,6,7].

The upcall of the North Atlantic right whale (*Eubalaena glacialis*, NARW) is a representative low-frequency contact call. This study focuses on NARW upcalls not only because they are ecologically and practically important detection targets, but also because they provide a methodologically useful benchmark. Compared with dolphin signature whistles or other marine mammal vocalizations with complex harmonic structures, NARW upcalls have a relatively clear upward frequency-modulated contour [8,9,10]. Even for this comparatively simple and structured call, however, it is not straightforward to determine automatically whether a sample produced by a generative model is a positive variation useful for detector training or a synthetic outlier that should be rejected. Conventional generated spectrograms do not provide a boundary for determining whether a generated sample is an outlier that is unlikely to help training. Therefore, NARW upcalls are an appropriate benchmark for formulating the problem of sample-level quality control in generative augmentation. If such QC is required even for this relatively simple upcall, the problem may become more severe for more complex bioacoustic signals involving harmonic structure, multi-component patterns, or individual signatures. From this perspective, this paper aims to propose a data augmentation approach that improves the detector decision boundary rather than simply expanding the number of samples.

Previous NARW upcall studies have reported that relatively simple augmentation methods such as SpecAugment and Mixup can improve precision and recall [11,12,13]. This indicates that augmentation is not merely an auxiliary technique but can have a substantial effect on detector generalization performance. However, conventional augmentation methods mainly focus on learning existing sounds and generating similar samples [11,12,13,14,15]. Therefore, in detector-oriented augmentation, the more important question is not how many samples can be generated, but which samples should be accepted and which should be rejected. For example, even if the duration of reference upcalls is generally distributed within 0.3–1.0 s, it is difficult to determine from a single feature alone whether a generated contour with a duration of 1.1 s is a plausible borderline variation useful for detector training, whether 1.2 s is still acceptable, or whether 1.33 s should be rejected as an outlier. Therefore, the quality of a synthetic positive sample should be evaluated not by a single feature threshold but within the joint distribution of multiple acoustic morphology features. In this study, QC does not mean discarding visually strange samples; rather, it is a numerical procedure for determining whether a generated contour lies within the morphology distribution of reference upcalls, whether a borderline deviation can be explained by uncertainty in the contour extraction process, or whether the sample should be rejected as a synthetic outlier for detector training.

This issue becomes more important when deep generative models such as Variational AutoEncoder (VAE), Generative Adversarial Network (GAN), and Denoising Diffusion Probabilistic Models (DDPM) are used. Previous bioacoustic augmentation studies have generated marine mammal vocalizations using waveforms, spectrograms, Mel-spectrograms, or latent representations and have shown that these synthesized samples can improve detection or classification performance [16,17]. In addition, DDPM models can generate high-dimensional spectrograms through iterative denoising and can therefore serve as strong generative augmentation baselines [16].

However, the fact that a generative model can produce samples that look or sound plausible does not mean that those samples should be used as positive training data. Existing generation-quality assessments often rely on reconstruction-level metrics such as Peak Signal-to-Noise Ratio (PSNR) and Structural Similarity Index Measure (SSIM), distribution-level metrics such as Frechet Inception Distance (FID) or Frechet Audio Distance (FAD), or perceptual evaluations such as Mean of Score (MOS) or visual inspection [11,12,13,14,15]. These metrics are useful for evaluating overall similarity or perceptual fidelity, but they do not directly indicate whether each synthetic sample is suitable for detector training, which acoustic features deviate from the expected range, or where rejection should begin. In particular, when waveforms or spectrograms are generated directly, target-call morphology is mixed with background noise, amplitude fluctuations, propagation effects, and time–frequency representation parameters, making it difficult to determine whether an abnormal generated sample reflects a plausible natural variation, a representation artifact, or a generative-model error.

To reduce these limitations, a QC-compatible representation is required, in which the generation result itself is interpretable, and the morphological characteristics of each sample can be computed directly. Therefore, this study uses the frequency contour of the NARW upcall as the generation target. The frequency contour directly represents the upward frequency trajectory that forms the core structure of the upcall. Thus, when the contour itself is the generation target, a generated sample can be converted directly into acoustic morphology features such as duration, frequency extent, sweep slope, and curvature, without having to interpret it from a complex signal representation.

Motivated by this perspective, this study proposes a VAE-QC framework that combines contour-level generation and morphology-based QC. In this framework, the 1D-VAE learns the variability of real upcall contours and generates synthetic contour candidates. Instead of adding all generated contours directly to detector training, each candidate is evaluated at the sample level to determine whether it has morphology suitable for detector learning. To this end, generated contour candidates are transformed into the same acoustic morphology feature space computed from real upcall contours. Each sample is evaluated by its multivariate deviation from the reference distribution, and only samples that pass the morphology consistency and uncertainty-aware acceptance criteria are used for final detector training. Therefore, the objective of this study is to select synthetic positives that are likely to be useful for detector training.

The proposed method uses the joint distribution of 10 features. Even if a sample has a slightly longer duration, it may be a plausible borderline variation if its frequency range, sweep rate, and curvature are consistent with real upcalls. Conversely, even if the duration is within the normal range, a contour may be a synthetic outlier unsuitable for detector training if its slope or curvature is abnormal. Therefore, the QC score in this study is used as a sample-level quality measure that considers the combination of the overall acoustic morphology rather than a single feature cut-off.

To train VAE-QC, a frequency-contour dataset extracted with high accuracy is required. Although several techniques can be used to extract upcalls, the currently most accurate method for frequency-contour extraction is direct human tracing [18,19,20]. However, uncertainty clearly remains even in manually extracted contours. This paper proposes a QC process that incorporates this uncertainty. To account for the uncertainty of the manual extraction process, this study empirically measures the magnitude of feature variations that can occur when the same individual repeatedly extracts the same upcall. If the deviation of a generated contour can be explained by this manual re-extraction uncertainty, the sample is allowed as a borderline case; otherwise, it is removed.

This design makes it possible to separately evaluate how well the intrinsic signal characteristics of generated samples are preserved and how useful the synthesized samples are for improving actual detector performance. Nevertheless, an improvement in detector performance alone does not necessarily mean that generated samples accurately reflect real upcall morphology. A deep detector may learn shortcut cues that happen to correlate with the training labels rather than the target signal structure. Therefore, this study applies QC to synthetic samples before detector training and then evaluates performance improvement under the same detector setting. In other words, the comparison is not between generator architectures alone, but between a spectrogram-level DDPM pipeline and a contour-level VAE-QC augmentation pipeline. Although DDPMs are strong baselines capable of generating high-dimensional spectrograms, they are costly in terms of sample-level morphology QC and computational expense. This study therefore adds a Contour-VAE without QC condition to separate the effect of contour generation from the effect of QC screening, and compares Original, DDPM, Contour-VAE without QC, and VAE-QC under the same detector and the same number of synthetic samples. Based on the above discussion, the main contributions of this study are as follows.

First, this study formulates NARW upcall generative augmentation not as a simple sample expansion problem, but as a detector-oriented quality control problem. Specifically, it addresses the sample-level decision of whether a generated positive sample represents a valid morphology variation that can be used for detector training or a synthetic outlier that should be rejected.

Second, this study proposes an uncertainty-aware QC criterion that combines a 10-dimensional acoustic morphology feature vector, a Mahalanobis-type QC score, and manual same-call re-extraction variability. This allows the core region of the real upcall distribution, the borderline region explainable by re-extraction uncertainty, and the synthetic outlier region to be numerically distinguished.

Third, this study compares Original, DDPM, Contour-VAE without QC, and VAE-QC under the same detector and augmentation budget to separate the effect of contour generation from the effect of QC screening. In addition, it analyzes the training time and sample generation time of Contour-VAE and DDPM to evaluate the computational cost of the proposed framework.

Fourth, the extracted upcall data and the re-extraction results used to measure the uncertainty introduced during manual re-extraction were made publicly available to support future research.

Therefore, this study is not a signal synthesis study that merely aims to generate more realistic whale sounds. Rather, it is a quality-controlled generative augmentation study that numerically determines which generated upcall samples are useful for detector training based on contour morphology and re-extraction uncertainty. Although the evaluation in this study is limited to the NARW upcall benchmark, it shows that sample-level QC is necessary even for a relatively simple upsweep structure and thus provides a starting point for quality-controlled augmentation of more complex bioacoustic signals.

## 2. Proposed Quality-Controlled VAE Augmentation Framework

### 2.1. North Atlantic Right Whale Upcall and Problem Formulation

The North Atlantic right whale (*Eubalaena glacialis*, NARW) produces various acoustic signals. Among them, the upcall is the representative call type most widely used in NARW PAM. The upcall is described as a stereotyped tonal upsweep signal whose frequency increases over time, and previous studies have reported that NARWs of all age classes and both sexes produce this signal. In particular, based on feeding-ground studies, Parks et al. described that all age and sex classes produce tonal stereotyped upsweep signals, namely upcalls, and that this signal is the most commonly used call type in NARW PAM [8,9,10].

**Figure 1:**
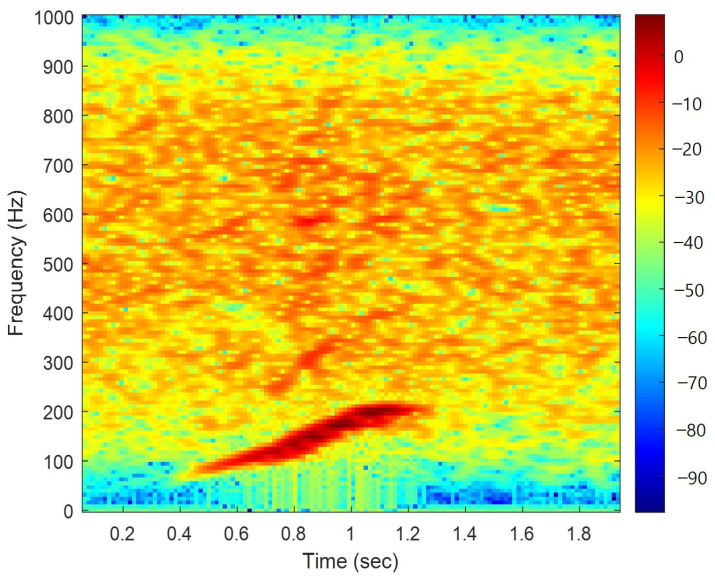
Example spectrogram generated by the authors from an audio segment in the NARW dataset [21].

This study focuses on NARW upcalls. Because they are not only important marker signals, but also because they provide a suitable benchmark for the sample-level quality-control problem. Compared with dolphin signature whistles or other marine mammal vocalizations with complex harmonic structures, NARW upcalls have relatively clear upward frequency-modulated contours. Accordingly, acoustic morphology such as duration, start/end frequency, frequency range, sweep rate, and curvature can be defined in a relatively interpretable manner. These properties make NARW upcalls an appropriate benchmark for formulating sample-level quality control of generated bioacoustic samples. Figure 1 shows an example spectrogram generated by the authors from an audio segment in the NARW dataset [21].

Acoustically, NARW upcalls have relatively simple upsweeping contours, but their call parameters cannot be represented by a single fixed value. Although upcalls generally have a low-frequency upsweeping FM structure, duration, start/end frequency, peak frequency, and bandwidth are random variables that vary within specific ranges depending on individuals, environments, and other factors [22]. This creates an important issue for quality control of generated samples: even when artificial signals are generated by an augmentation method, the boundary for determining whether a generated sample will help improve detector performance is unknown. Therefore, even for the relatively simple NARW upcall, a numerical procedure is needed to determine whether a generated sample is a valid positive variation or a synthetic outlier that should be rejected.

The same issue arises in detector-oriented generative augmentation. When adding synthetic positive samples to detector training, the important question is not how many samples are generated, but whether each generated sample lies within the possible morphology variation of real upcalls and is likely to help detector learning. If a generated sample is a synthetic outlier that deviates substantially from real upcall morphology, including it with a positive label can distort the detector decision boundary.

The previous studies and detector designs described above are directly related to the problem formulation of this study. The reason this study represents upcalls not as full raw waveforms but as time-varying frequency trajectories, that is, frequency contours, is not simply to reduce signal dimensionality. The frequency contour directly reflects the core upsweeping FM morphology of an upcall and can be transformed into interpretable acoustic features such as duration, frequency range, sweep rate, and curvature. Therefore, the contour representation is a QC-compatible representation that enables generated samples to be inspected and selected at the sample level before detector training.

In detector-oriented augmentation, the key question is whether a generated contour lies within the possible morphology variation of real upcalls or should be removed as a synthetic outlier before detector training. For example, even if the duration of reference upcalls is generally distributed within a certain range, it is difficult to determine from duration alone whether a slightly longer contour is a plausible borderline variation or an outlier that should be rejected. A somewhat longer duration may still be useful for detector training if the frequency range, sweep rate, and curvature are consistent with real upcalls; conversely, a contour with a normal duration may be unsuitable as a positive training sample if its slope or curvature is abnormal. Therefore, the quality of a generated upcall should be evaluated within the joint distribution of multiple morphology features rather than by a single acoustic parameter. From this perspective, this study represents NARW upcalls using the following narrowband amplitude-frequency modulated signal model.

(1) $$x\left(t\right)=a\left(t\right)cos\left(\phi_{0}+2\pi t\int_{0}^{t}f(\tau )d\tau \right).$$

Here, $x\left(t\right)$ is the upcall waveform, $a\left(t\right)$ is the amplitude modulation term, $f\left(\tau \right)$ is the frequency contour, and $\phi_{0}$ is the initial phase. The frequency contour $f\left(\tau \right)$ represents the core time-varying frequency trajectory of the upcall and is the generation and QC target in this study. Therefore, the generation problem is not defined as direct generation of the full waveform $x\left(t\right)$, but as learning the contour characteristics that constitute the structural component of the upcall and using them to construct synthetic upcalls for detector training. In this study, $a\left(t\right)$ is implemented using smooth amplitude modulation based on duration-matched Gaussian or Hamming windows. The QC procedure does not evaluate amplitude realism; rather, it evaluates whether the acoustic morphology of a generated contour is suitable for detector training.

The most accurate current approach for extracting whale upcall frequency contours is to visually inspect the spectrogram and trace the contour manually [18,19,20]. However, even manual extraction introduces uncertainty in contour-based representations. Even when the same analyst extracts the same call repeatedly, the contour and feature values can vary depending on STFT parameters, time alignment, background noise, SNR, and extraction conditions. Therefore, it is not appropriate to remove all generated contours that slightly deviate from the real distribution boundary as synthetic outliers. Some samples may fall within the range of uncertainty that can occur during actual contour extraction and thus represent plausible borderline variations.

For this reason, this study does not use the contour merely as a generation target. Instead, it performs quality control of generated samples using both acoustic morphology features extracted from the contour and same-call re-extraction variability. The proposed VAE-QC framework numerically determines whether a generated contour lies in the core region of the reference upcall distribution, in the borderline region explainable by re-extraction uncertainty, or in the synthetic outlier region that should be rejected for detector training. This is the core of the VAE-QC augmentation framework proposed in the next section.

### 2.2. Contour 1D-VAE Generation

This study proposes a VAE-QC augmentation framework to improve North Atlantic right whale upcall detection performance. Instead of directly generating the entire raw waveform of the upcall, the proposed method models the frequency contour defined in Section 2 as a one-dimensional time series. However, not every candidate generated by the VAE is suitable for detector training. Some generated contours may represent rare natural variations of upcalls observed in real datasets, whereas others may be abnormal shapes caused by latent sampling, decoder reconstruction error, or instability in the manual contour tracing process. Therefore, this study screens generated contours by jointly considering the real upcall feature distribution and the manual re-extraction variability of the same call. In other words, the key idea of the proposed method is not a simple VAE generator, but a detector-oriented augmentation framework that integrates generation, re-extraction uncertainty calibration, and feature-based screening.

As defined in Section 2.1, a NARW upcall can be represented by a frequency contour. The reference contours used in this study were not extracted by an automatic algorithm; instead, they were obtained by manually tracing the dominant upsweeping ridge of each upcall. For notational simplicity, let the contour of the *i*-th upcall be $f_{i}$. Here, $f_{i}$ is a time-series vector normalized to size 1 × *L*. In this study, the contour is learned using a 1D-VAE. The VAE consists of an encoder and a decoder. The encoder compresses the input time series into a latent vector, and the decoder reconstructs the time series from this latent vector. This procedure can be written as follows.

(2) $$enc\left[f_{i}\right]\to z_{f}\to dec\left[z_{f}\right]\to {\hat{f}}_{i}$$

In Equation (2), $enc\left[\cdot \right]$ and $dec\left[\cdot \right]$ denote the encoder and decoder, respectively. $z_{f}$ denotes the latent vector, and ${\hat{f}}_{i}$ denotes the generated contour time series. Through this procedure, the VAE learns the contour variations present in real upcall data, and the generated contour is then converted into a synthetic waveform according to the upcall model in Section 2 as follows.

(3) $$\hat{x_{i}}\left(t\right)=a_{i}\left(t\right)cos\left(\phi_{0}+2\pi t\int {\hat{f}}_{i}(t)dt\right).$$

Here, ${\hat{f}}_{i}\left(t\right)$ is the frequency contour generated by the VAE, $a_{i}\left(t\right)$ is the amplitude modulation term based on a duration-matched Gaussian or Hamming window, and $\phi_{0}$ is the initial phase. An important advantage of using a contour representation in this study is that quality control can be performed directly on the generation results. If a generative model directly generated the entire raw waveform or spectrogram, contour extraction would have to be performed again to determine whether the generated signal follows real upcall morphology, and acoustic features would then need to be extracted from it. In that case, generation quality evaluation would again depend on the accuracy of an additional contour extraction process, making it difficult to distinguish generation errors from manual re-tracing variability.

In contrast, VAE-QC generates the frequency contour ${\hat{f}}_{i}\left(t\right)$, which represents the core structure of the upcall, rather than the raw waveform itself. Therefore, a QC feature vector can be computed directly from the generated contour and immediately compared with the feature distribution obtained from real upcall contours for screening. In other words, the proposed method does not generate a synthetic waveform and then re-extract signal characteristics for verification; instead, it already provides a structurally QC-compatible representation at the generation stage, which is advantageous for quality control. Thus, the contour representation is a QC-compatible representation that allows generation results to be directly converted into the QC feature space. However, the fact that a generated contour can be directly evaluated in the QC feature space does not mean that all generated contours are suitable for detector training. Some generated contours may correspond to rare but plausible morphology variations that can occur in real data, whereas others may be synthetic outliers that deviate from real upcall morphology because of latent sampling, decoder reconstruction limitations, or instability in the contour generation process. The next section describes how these candidates are distinguished using feature-based QC and re-extraction variability.

### 2.3. Morphology Feature Computation and Manual Re-Extraction Variability-Based QC

To evaluate generated contours, this study extracts a 10-dimensional acoustic morphology feature vector from each contour. These features include the linear trend, peak structure, start/end frequency, local slope, duration, and frequency range of the contour, and they summarize the morphology of the upcall contour in an interpretable form. The full feature list is provided in Table 1.

The detailed procedure for extracting the features in Table 1 is described in Appendix A. Finally, let F denote the feature vector extracted from contour $f_{i}$. This feature vector serves as the feature representation used to determine whether generated contours are outliers.

To determine whether a VAE-generated contour is out-of-distribution, a reference real upcall feature distribution is first required. This study uses real upcall contours manually traced while visually inspecting the spectrogram as the reference set. Ten-dimensional feature vectors are then computed from these manually extracted real contours, and the reference acoustic morphology distribution is constructed in the feature space.

Let $f_{i}^{real}$ be an arbitrary manually extracted real upcall contour, and let $F_{real}$ be the feature set extracted from all real upcall contours. Similarly, let ${\hat{F}}_{i}$ be the feature set extracted from a generated contour ${\hat{f}}_{i}$. Whether the generated contour is an outlier can then be determined by assessing how far ${\hat{F}}_{i}$ deviates from $F_{real}$. When determining whether a generated contour is an outlier, both feature-wise scale differences and correlations among real upcall features must be considered. Therefore, this paper uses the Mahalanobis distance. Finally, the Mahalanobis distance for QC of the generated contour ${\hat{f}}_{i}$ is computed as in Equation (4).

(4) $$s\left({\hat{f}}_{i}\right)={\left(R\left({\hat{F}}_{i}\right)-\mu_{real}\right)}^{T}S_{real}^{-1}\left(R\left({\hat{F}}_{i}\right)-\mu_{real}\right),$$

In Equation (4), $R\left(\cdot \right)$ denotes robust normalization based on the median and Interquartile Range (IQR) of the real feature set, which reduces feature-wise scale differences. $\mu_{real}$ and $S_{real}$ denote the mean vector and regularized covariance matrix of the normalized real upcall feature distribution, respectively. Therefore, $s\left({\hat{f}}_{i}\right)$ indicates how far the features of a generated contour generated by the VAE deviate from the real upcall feature distribution. A small score means that the sample lies close to the real upcall feature cloud, whereas a large score indicates that the contour may be difficult to explain by real upcall morphology. Therefore, a QC threshold is required to determine whether a generated contour should be retained as a normal synthetic sample or removed as an out-of-distribution sample. In this study, the reference feature distribution was defined using the Mahalanobis-type QC scores of the feature vectors from the manually extracted real upcall contours.

(5) $$\tau_{\alpha }^{data}=Q_{\alpha }\left(s\left(f_{i}^{real}\right)\right).$$

In Equation (5), $Q_{\alpha }\left(\cdot \right)$ denotes the empirical α-quantile, calculated from the sample dataset, i.e., the value below which the lower α fraction of the data lies. The threshold $\tau_{\alpha }^{data}$ in Equation (5) is a hard threshold that defines the basic range regarded as normal morphology in the real upcall feature distribution. Therefore, if the score of a generated contour falls within this boundary, the sample is always accepted. However, removing all samples that slightly exceed the hard threshold can be overly strict.

Even for the same upcall, the contour and its feature values can vary slightly depending on spectrogram display conditions, STFT parameters, time–frequency resolution, background noise, SNR, and the process by which an analyst traces the ridge. In other words, it is necessary to estimate the feature-level uncertainty that may arise when the same upcall is re-traced and incorporate it into the screening criteria. For this reason, this study does not remove all generated contours that exceed the hard threshold. If a sample slightly exceeds the hard threshold but falls within the range explainable by actual manual re-extraction variability, it is regarded as a plausible borderline variation; otherwise, it is removed as a synthetic outlier. If the feature vector from the first contour extraction of an upcall is $F_{real}^{\left(0\right)}$ and the feature vector obtained under the m-th re-extraction condition is $F_{real}^{\left(m\right)}$, the score variability is defined as follows.

(6) $$\Delta s_{i}^{\left(m\right)}=\left|s\left(F_{real}^{\left(m\right)}\right)-s\left(F_{real}^{\left(0\right)}\right)\right|.$$

The maximum score variability among multiple manual re-extraction conditions is then computed as follows.

(7) $$\Delta s=\underset{m}{max}\Delta s_{i}^{\left(m\right)}.$$

Here, Δs represents score variability caused by manual uncertainty occurring during the analysis process. In this paper, Δs is used to define the width of the borderline region. For example, suppose $\tau_{\alpha }^{data}$ is 30 and Δs is 10. A generated contour with a score of 30 or less is always accepted, whereas a generated contour with a score greater than 40 is always rejected. A generated contour with a score between 30 and 40 is regarded as a borderline sample, and in this region the acceptance decision is made probabilistically based on how often similar score exceedances occurred in the re-extraction experiment.

The hard threshold exceedance of a generated contour is defined as $d_{i}=s\left({\hat{f}}_{i}\right)-\tau_{\alpha }^{data}$. $A_{out}\left(d_{i}\right)$ is an empirical acceptance function calibrated from same-call manual re-extraction variability. In other words, when the generated contour slightly exceeds the hard threshold, we first verify whether the magnitude of this excess falls within the range of variation observable when a human re-traces the same call; we then use the empirical probability of such an excess occurring as the acceptance probability. The specific calibration of $A_{out}\left(d_{i}\right)$ is described in Section 3.1. Based on this definition, the acceptance probability of a generated contour is defined as follows.

(8) $$P_{acc}\left({\hat{f}}_{i}\right)=\left\{\begin{array}{c} 1,\;\\ A_{out}\left(d_{i}\right),\\ 0,\; \end{array}\;\right.\begin{array}{c} \;s\left({\hat{f}}_{i}\right)\leq \tau_{\alpha }^{data}\;\\ \tau_{\alpha }^{data}<s\left({\hat{f}}_{i}\right)\leq \tau_{\alpha }^{data}+\Delta s\\ \tau_{\alpha }^{data}+\Delta s<s\left({\hat{f}}_{i}\right)\; \end{array}$$

**Figure 2:**
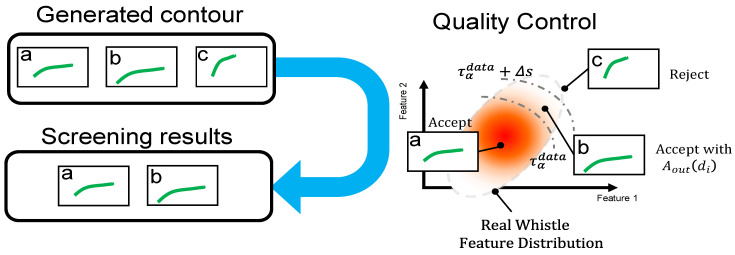
Concept of the proposed QC-based contour screening procedure.

That is, generated contours inside the hard threshold are always accepted; within the borderline region explainable by manual re-extraction uncertainty, generated contours are accepted probabilistically with manual re-extraction uncertainty. Generated contours outside the borderline region are always rejected. This process is illustrated in Figure 2.

**Figure 3:**
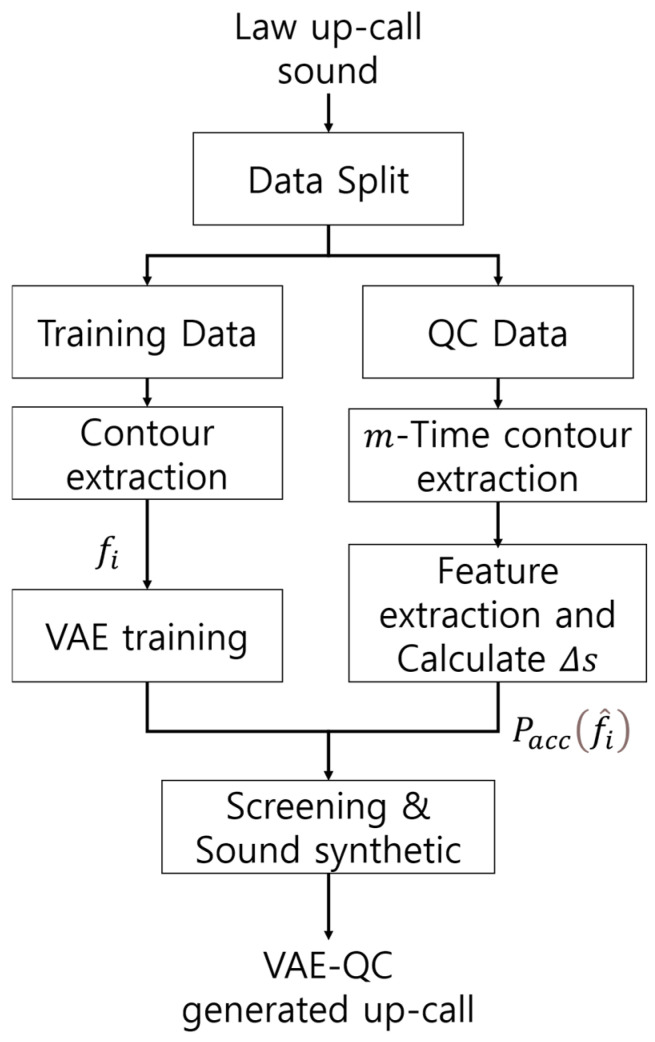
Overall workflow of VAE-QC training, screening, and synthetic upcall generation.

In Figure 2, three upcall contours, a, b, and c, are generated by the VAE contour generator. At first glance, these contours may appear similar to upcalls, but it is not possible to determine visually how similar to real upcalls or whether they sufficiently reflect the characteristics of up. Therefore, the proposed quality control method screens each contour using a Mahalanobis-type distance to measure how similar it is to the feature distribution of real upcalls, and only accepted contours are used. In this process, Equation (8) incorporates uncertainty that arises during manual contour extraction. Thus, the proposed VAE-QC is not a simple outlier rejection method, but a quality-controlled augmentation framework that jointly controls the morphology consistency of synthetic contours and the diversity of positive variation required for detector training, based on the morphology distribution of manually traced real upcall contours and manual re-extraction uncertainty. The proposed synthetic upcall training and generation procedure is shown in Figure 3.

As shown in Figure 3, the proposed method first learns contours, screens them using scores computed from the extracted features, and then generates artificial signals. This is advantageous for detector training in two respects. First, it reduces the possibility that abnormal contours generated by the VAE or artifacts that are difficult to explain by real upcall morphology are included in detector training. Second, even when a sample slightly exceeds the hard threshold, it is selectively allowed if the deviation can be explained by variability observed during same-call re-extraction, thereby avoiding excessive removal of rare real upcall variations. The next section experimentally analyzes how well this QC-based synthetic set matches the real feature distribution and how it contributes to detector performance.

## 3. Experimental Results and Analysis

This section evaluates the proposed VAE-QC framework from the perspective of detector-oriented generative augmentation. The objective of this study is whether quality control of generated samples actually contributes to detector performance improvement through augmentation under the same NARW upcall benchmark, the same detector, and the same synthetic-sample budget.

The experiments consist of three stages. First, the QC threshold and the borderline acceptance probability are calibrated using manual re-extraction variability of real upcall contours extracted by human analysts. Second, the feature distributions of generated contours before and after QC are compared with the real upcall distribution to evaluate the morphology consistency of the final synthetic set. Finally, the effects of QC-based sample selection on detector performance are analyzed by comparing Original, DDPM, Contour-VAE without QC, and VAE-QC under the same detector setting.

The experiments used a total of 30,000 acoustic segments provided by Kaggle [21]. Of these, 7027 are positive samples containing NARW upcalls, and 22,973 are negative samples corresponding to non-upcalls or background. The experimental design of this study focuses on analyzing, in a controlled setting, how quality-controlled augmentation affects feature-space consistency and detector-oriented utility, rather than on noise-specific robustness or cross-dataset generalization.

### 3.1. QC Calibration from Re-Extraction Variability

Training VAE-QC requires a frequency-contour dataset of upcalls extracted with high accuracy. Although several methods can be used to extract upcalls, the most accurate current approach for frequency-contour extraction is direct human tracing [18,19,20]. In addition, previous whale whistle studies have manually extracted time–frequency contours from spectrograms to analyze acoustic characteristics [23]. Following this approach, we manually extracted the time–frequency contours of NARW upcalls. A MATLAB (v2026)-based contour-extraction UI was used [23]. In this study, extraction was performed for all 7027 NARW upcalls. To measure manual re-extraction uncertainty, 514 of these upcalls were re-extracted 4 times. Among these extraction results, the time–frequency contours of 6000 upcalls have been made publicly available. In addition, the time–frequency contours obtained from the re-extraction process have also been released. The resulting re-extraction data were used as the uncertainty calibration criterion for judging borderline generated samples around the hard threshold.

**Figure 4:**
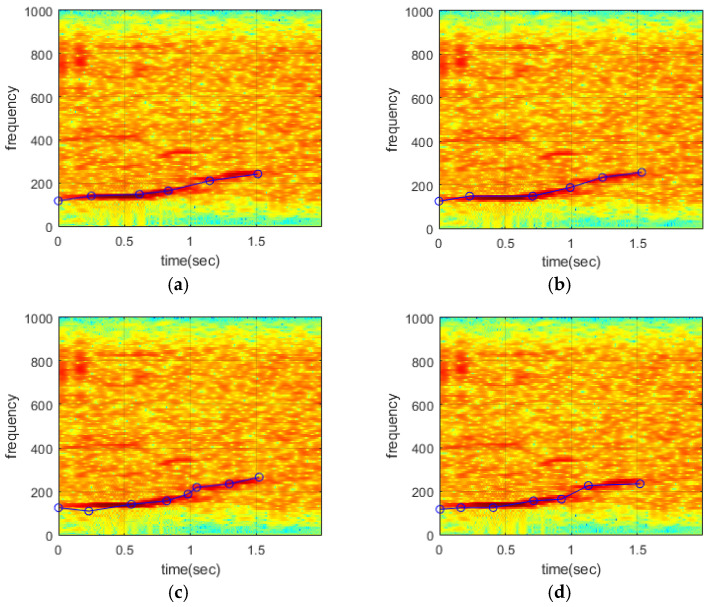
Example upcall contour manual re-extraction results: (**a**) FFT 256, overlap 250; (**b**) FFT 512, overlap 500; (**c**) frequency reversal; and (**d**) FFT 256, overlap 250.

In the QC calibration, re-extraction variability was measured by applying different contour extraction conditions to the same upcall. In this study, extraction was repeated four times. Thus, m in Equations (6) and (7) is four. During re-extraction, the STFT parameters were changed once, the frequency axis was reversed once, and the final extraction was repeated under the same conditions as the initial extraction. The following Figure 4 shows examples of the re-extraction results. In Figure 4, the blue line represents the result of contour extraction performed by a human.

**Figure 5:**
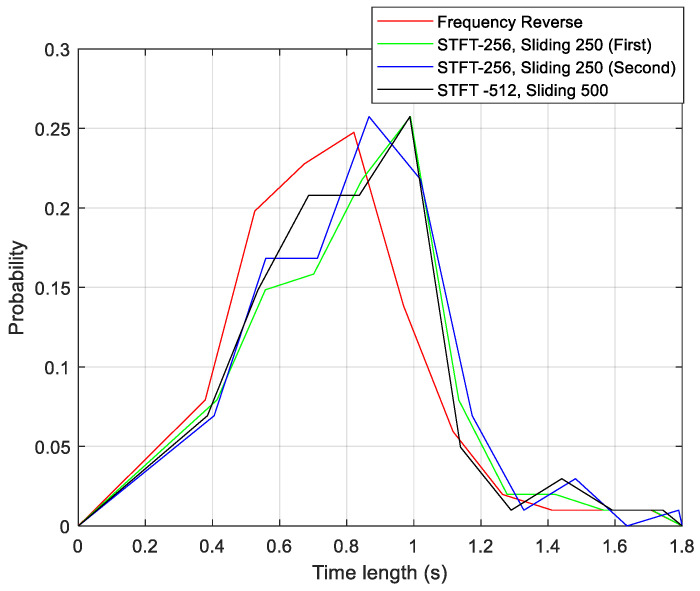
Distribution of time duration obtained from same-call manual contour re-extraction.

Figure 4 shows that changing the extraction conditions, as in (b) and (c), produces slight differences in the extraction results. In particular, although (a) and (d) were used to measure differences when the extraction was repeated with the same parameters, slight differences still occurred. These differences do not reflect differences in the signal itself; rather, they represent variability that can occur during contour extraction and serve as the basis for handling borderline samples near the QC threshold. Therefore, even if a generated contour slightly exceeds the hard threshold of the real feature distribution, it should not be immediately regarded as an abnormal sample; instead, it is necessary to check whether the deviation can be explained by the variability observed in same-call re-extraction. To examine this variability, we analyzed the distributional variability of the signal features obtained from the extraction results. Figure 5 shows the time-duration distribution of the extraction results.

Figure 5 shows that features extracted from a contour can vary depending on the re-extraction condition. Accordingly, features representing the characteristics of the upcall can exhibit some variability based on the manual re-extraction uncertainty. The feature variability is summarized in Table 2. This table shows how much the ten contour features can change on average during re-extraction. In particular, features such as slope, maximum slope, and duration show relatively large variability depending on the extraction condition, which makes them important factors to consider when interpreting samples that slightly exceed the hard threshold during QC.

**Figure 6:**
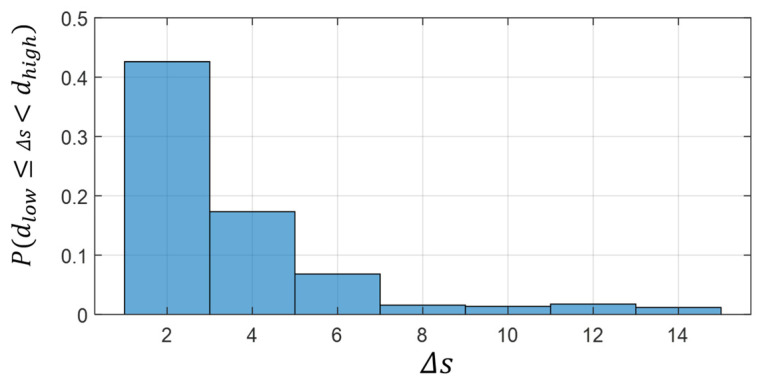
Empirical distribution of outward QC score shifts obtained from same-call manual contour re-extraction.

Based on the manual re-extraction uncertainty of the ten features in Table 2, Delta s was calculated to be approximately 15.78. Using this re-extraction uncertainty, the borderline region around the hard threshold $\tau_{\alpha }^{data}$ was defined, and the acceptance probability was computed at each percentile position. Specifically, among the 514 samples, the Mahalanobis-type QC score containing 97% of the data was 4.1, and the score containing 90% of the data was 3.1. Thus, $\tau_{\alpha =0.97}^{data}$ is 4.1 and $\tau_{\alpha =0.9}^{data}$ is 3.1. If one wants to use only contours corresponding to typical upcall shapes, that is, contours lying within 90% or 97% of the feature distribution, all contours with Mahalanobis-type scores exceeding 3.1 or 4.1 could simply be rejected. However, not all contours that slightly exceed the hard threshold are necessarily unrealistic outliers. Some contours may represent rare but plausible morphology variations observed in real data, and such samples may help expand positive variation during detector training. To evaluate this possibility, this study uses the QC score-shift distribution observed in same-call manual re-extraction to determine whether the hard threshold exceedance can be explained by manual annotation uncertainty. The Δs and $A_{out}\left(d_{i}\right)$ values obtained through the manual re-extraction process shown in Figure 4 are summarized in Figure 6.

Figure 6 shows the empirical distribution of outward QC score shifts Δs obtained from same-call manual contour re-extraction. The acceptance probabilities in Table 3 and Table 4 were computed by integrating the probability mass of the $d_{i}$ intervals defined for each hard threshold setting from this distribution. Because the QC score is a Mahalanobis-type distance, its absolute unit does not have a direct physical meaning like an acoustic parameter. For example, an increase in the value $s\left(f_{i}^{real}\right)$ from 4 to 5 does not necessarily imply the same acoustic rarity as an increase $s\left(f_{i}^{real}\right)$ from 15 to 16. Therefore, in this study, the intervals were defined based on the QC score percentile boundaries of manually extracted real upcall contours. In other words, the $d_{i}$ intervals were determined according to the percentiles of the QC score ($Q_{\alpha }\left(s\left(f_{i}^{real}\right)\right)$), and the acceptance probability for each interval was obtained by integrating the probability in Figure 6. The resulting values are shown in Table 3 and Table 4.

In the next section, the manually extracted contours are used as training data to train the VAE and generate synthetic contour candidates. We then evaluate whether the generated contours have acoustic morphology feature distributions similar to those of manually extracted real upcall contours and whether these characteristics contribute to improved detector performance.

### 3.2. Feature-Space Effects of QC-Based Synthetic Sample Selection

Before evaluating the performance of the proposed method, we describe the training process and data generation procedure. To analyze how the performance of VAE-QC changes according to the threshold value that determines QC control, experiments were conducted for the cases of α = 0.90 and α = 0.97, as shown in Table 3 and Table 4, as well as for the case without QC screening. In addition, performance was compared with DDPM, which is known to perform well for single whale-call augmentation among generative methods [16]. The resulting synthetic sets were then evaluated from two perspectives. First, we examined whether QC screening actually changes the acoustic morphology distribution of the generated contours in the intended direction. Second, we evaluated whether this feature-space screening is actually beneficial for detector training. To evaluate these aspects, 300 synthetic candidates were generated for each method. In the VAE-QC condition, 300 samples that passed QC were used, whereas in the Contour-VAE without QC and DDPM conditions, the same number of generated samples was used. The training data used for the VAE-QC were manually extracted upcall time–frequency contours. The contours used to measure manual re-extraction uncertainty were excluded from VAE-QC training. For a fair comparison, the DDPM model was trained using the same number of upcalls spectrogram. The number of samples used to train each generator is shown in Table 5.

**Figure 7:**
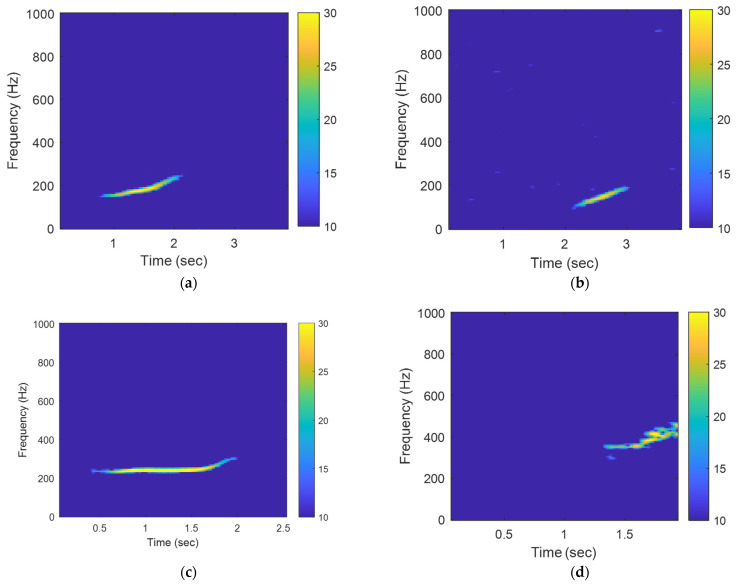
Examples of synthetic upcall spectrograms generated by each augmentation method: (**a**) VAE-QC α = 0.97, (**b**) VAE-QC α = 0.90, (**c**) Contour-VAE without QC, and (**d**) DDPM.

In this study, contours were generated using a 1D convolutional VAE, and an unconditional DDPM was constructed as the comparison model. The VAE used 128-point contours extracted from denoised signals as inputs. The encoder converted the input contour into the mean and variance of the latent distribution through stride-2 convolution layers, and the latent vector was sampled using the reparameterization trick. The decoder was trained to reconstruct the 128-point contour from the latent vector. The training loss was defined as the sum of the reconstruction MSE and KL divergence. After training, synthetic contour candidates were generated by sampling from the latent prior. The QC rule defined in Section 3.1 was then applied. The selected contours were then used to generate time-series signals, and smooth amplitude modulation based on duration-matched Gaussian or Hamming windows was applied to construct the waveforms. A total of 300 signals were generated for each method, and Figure 7 shows example spectrograms of the generated upcalls.

**Figure 8:**
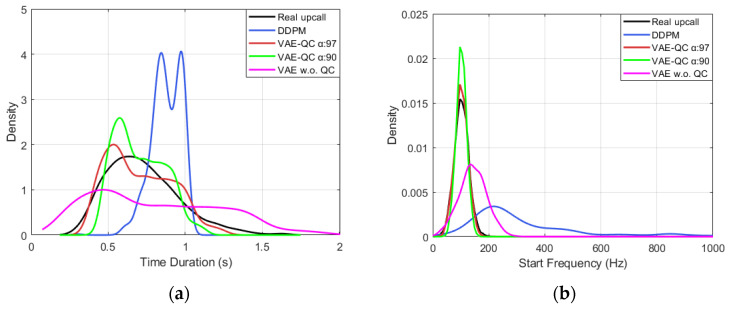
Example feature distributions from real and generated upcall contours: (**a**) time duration and (**b**) start frequency.

Figure 7 shows example spectrograms of synthetic upcalls generated by each augmentation method. The VAE-QC conditions in Figure 7a,b are results reconstructed into time-series signals from QC-passed contours, and they show relatively clear upcall-like contours whose frequency increases over time in the low-frequency band. Because the generated contours in both cases passed QC screening, their duration and frequency trajectories are constrained to lie within the allowable range of the reference upcall morphology. In contrast, the Contour-VAE without QC example in Figure 7c was generated by the same contour VAE but without QC screening. This example includes a partially upcall-like upsweep structure, but it also indicates that contour duration and frequency trajectory can vary more widely than in the VAE-QC samples. To examine whether the proposed QC procedure adjusted the morphology of the generated samples in the intended direction and to compare it with other methods, we analyzed the feature distributions and QC scores of 300 synthetic up-calls generated by each method. Because DDPM directly generates spectrograms, it is difficult to extract acoustic features directly from signal contours. Therefore, in this study, as in the feature extraction process for real up-calls, the up-call contours were manually extracted from 300 DDPM-generated spectrograms [23]. Although the proposed QC is performed based on the full 10-dimensional feature vector, the results for two representative features, time duration and start frequency, are presented in Figure 8 as examples.

Figure 8a shows the time duration distribution, and Figure 8b shows the start frequency distribution. Real upcall contours are mainly distributed at durations of approximately 0.4~1.1 s and start frequencies of approximately 100–160 Hz. In contrast, Contour-VAE without QC has a much wider duration range of approximately 0.1~2.0 s, includes a long-duration tail above 1.5 s, and shows a broader start frequency distribution shifted toward higher frequencies than the real distribution. DDPM samples are concentrated in a relatively narrow duration range, but their start frequencies include a high-frequency tail extending beyond 200 Hz and, in some cases, above 500 Hz.

**Figure 9:**
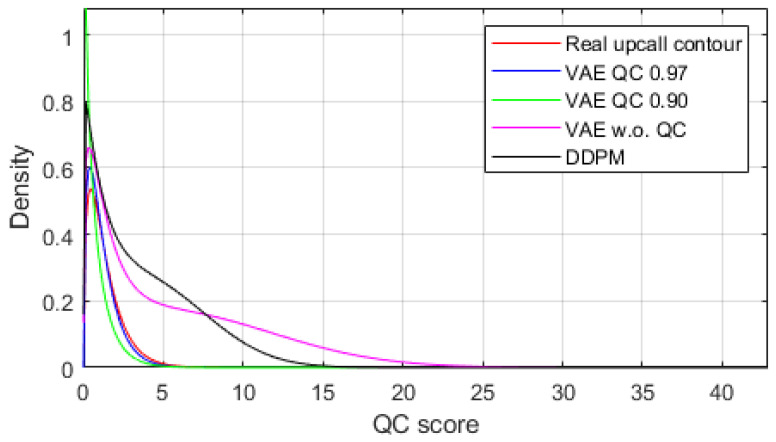
QC score distributions of real calibration contours and generated samples.

VAE-QC suppresses these feature-level outliers and restricts the duration and start frequency of the final synthetic set to ranges closer to the real upcall distribution. In particular, the α = 0.97 condition preserves a wider range of real-like positive variation than the α = 0.90 condition while removing the extreme outliers observed in Contour-VAE without QC. This shows the necessity of morphology-based thresholding before generated samples are used for detector training. However, because sample validity cannot be determined solely from duration or start frequency, this study also computed the distribution of Mahalanobis-type QC scores obtained from the 10-dimensional feature space, as shown in Figure 9.

Figure 9 compares the Mahalanobis-type QC score distributions of manually extracted real upcall contours and generated samples. Here, the real upcall contour score distribution, shown as the red curve, serves as the reference baseline for evaluating generated samples. As a result, both VAE-QC α = 0.90 and α = 0.97 are concentrated in the low-score region where the real calibration contours are mainly located, and they substantially suppress the high-score tails observed in Contour-VAE without QC and DDPM. In particular, VAE-QC α = 0.90 shows the most conservative score distribution, whereas α = 0.97 allows a wider range of positive variation while still limiting the high-score outlier tail. This indicates that the QC rule defined above does not simply use more generated samples, but operates to construct a synthetic set more consistent with the real upcall morphology distribution. However, improved feature distribution alone does not guarantee improved detector performance. Therefore, the next section compares Original, DDPM, Contour-VAE without QC, and VAE-QC under the same detector and augmentation budget to determine whether threshold-based QC leads to actual detector performance improvement.

### 3.3. Detector Performance with Quality-Controlled Augmentation

Detector performance was evaluated to determine whether quality-controlled augmentation is useful for actual detector training. In this study, we did not arbitrarily design a new detector architecture. Instead, we used a publicly available STFT-CNN-based whale detector for the Kaggle whale detection challenge as the downstream detector [24]. The repository uses a structure that converts 1D acoustic signals into STFT time–frequency representations and then performs binary whale-signal detection using an Inception-V1 CNN architecture. To train this detector, the entire dataset was independently randomly split three times.

As shown in Table 6, the data assigned to Subset 1 in each split were used to train the generator and the detector. Subset 2 was used to evaluate the detection performance of the trained detector. This separation was used to ensure that detector evaluation data were not used during generator training or QC calibration. The data composition for each split is shown in Table 6. The data used to train each generator were constructed from Subset 1, as shown in Table 5.

To evaluate how effectively synthetic upcalls generated from the trained generators improve detector performance, the detector training conditions were defined as Original, DDPM, Contour-VAE without QC, and VAE-QC. The Original condition used only Subset 1 data without synthetic samples. The DDPM condition added 300 synthetic upcall spectrograms generated by the DDPM. The Contour-VAE without QC condition added 300 samples generated by the Contour-VAE without QC screening. The VAE-QC condition added 300 synthetic samples that passed the proposed QC.

Unlike DDPM, which directly generates spectrograms that include background texture, the Contour-VAE-based methods reconstruct clean tonal signals from frequency contours. Therefore, artificial noise must be inserted. Previous work has noted that directly mixing real background noise obtained under the same environment as the evaluation data into synthetic training samples can lead to overestimated detector performance [16]. Therefore, this study did not reuse evaluation backgrounds directly. Instead, independent Gaussian noise was inserted according to the mean and range of SNR values measured from Subset 1 data. This procedure was not a noise-type-specific robustness experiment, but a controlled waveform reconstruction step for constructing contour-based synthetic signals at a noise level suitable for detector input. The same procedure was applied to both Contour-VAE without QC and VAE-QC; therefore, the difference between these two conditions is limited to whether QC screening was applied. All other conditions were kept the same. The data composition used for detector training is shown in Table 7.

**Figure 10:**
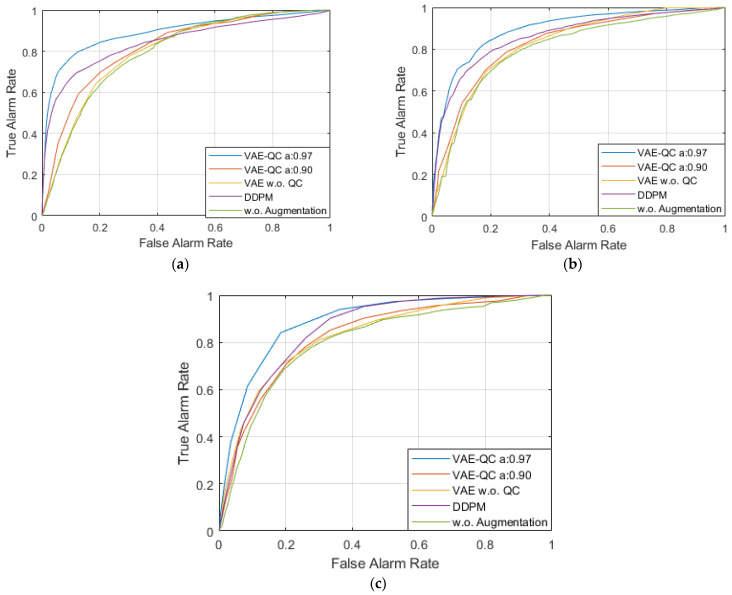
ROC curves for detector performance under each augmentation condition: (**a**) split 1, (**b**) split 2, and (**c**) split 3.

For each random split, the above training configuration was repeated three times. Therefore, for each method, detector performance was obtained over 3 splits × 3 repeated runs, yielding 9 results per method. Across the 5 detector conditions, a total of 45 detector performance results were obtained. Among these, the learning and detection results from the first run of each split are shown in Figure 10. To summarize the performance of the 45 detectors by method, Table 8 reports the mean and standard deviation over 3 splits × 3 repeated runs for each method.

Table 8 shows the mean and standard deviation of detector AUC obtained from three random splits and three repeated training runs for each split. The Original condition without augmentation achieved a mean AUC of 0.802. The proposed VAE-QC α = 0.97 condition achieved the highest mean AUC among all conditions, 0.901, corresponding to improvements of +0.099 over Original, +0.056 over DDPM, and +0.091 over Contour-VAE without QC. This result highlights the importance of defining which feature ranges should be accepted as positive variations useful for detector training.

Contour-VAE without QC achieved a mean AUC of 0.810, only a slight improvement over the Original AUC of 0.802, indicating that contours generated without QC may include morphology outliers unsuitable for detector training. Conversely, VAE-QC α = 0.90 achieved a mean AUC of 0.824, higher than Original but lower than DDPM, suggesting that an overly conservative threshold may remove not only outliers but also plausible positive variations useful for detector learning. DDPM improved performance with a mean AUC of 0.845; however, as shown by the preceding feature and QC-score distributions, it still included some high-score or outlier tails. This suggests that a certain level of feature deviation may help detector learning, but that performance improvement can be limited if the allowable range is not controlled. In addition, because DDPM performs spectrogram-level generation, its performance may have partially benefited from learning the background/noise texture of the same benchmark.

The ROC curves in Figure 10 are consistent with Table 8. Across the three splits, VAE-QC α = 0.97 showed the highest true alarm rate over most false alarm rate ranges. Therefore, the detector-performance results can be interpreted as showing that the proposed QC does not simply use more synthetic samples or remove samples too strictly; rather, it sets feature boundaries that are useful for improving detector performance based on the real upcall morphology distribution and manual re-extraction variability. In other words, these results show that an important factor in generative augmentation is not only the generator itself, but also data-driven QC thresholding that determines which acoustic morphologies should be admitted into detector training. The detector performance results further indicate that the proposed VAE-QC has practical advantages as an augmentation pipeline, not only in terms of detection performance. To demonstrate this, Table 9 shows the mean values obtained when generating 300 samples.

As shown in Table 9, DDPM is a strong baseline that directly generates spectrogram-level samples, but its iterative denoising process requires high training and sample generation costs. In this experiment, DDPM required approximately five days for generator training and approximately 80 s to generate one sample using 300-step sampling. In contrast, the Contour-VAE required approximately two days for training, approximately 0.31 s for contour generation per sample, and approximately 2.01 s for end-to-end sample construction, including QC and waveform/spectrogram reconstruction. Therefore, VAE-QC can generate many candidate contours at substantially lower generation cost than DDPM and select samples suitable for detector training through QC. This shows that improving detector performance does not necessarily require a complex spectrogram-level generator; a lightweight VAE-based pipeline can also obtain high detector utility when the core morphology of upcalls is modeled as contours and feature-based QC is applied.

## 4. Discussion

This study proposed a VAE-QC augmentation framework for NARW upcall detection. The central objective of this study was not to synthesize NARW upcalls more realistically, but to formulate the quality control problem of deciding which synthetic positive samples should be used for detector training. Increasing the number of samples in generative augmentation is relatively easy, but whether generated samples actually help detector training is a separate question. The key is to define, in a data-driven manner, the acceptance boundary between positive variations useful for detector learning and synthetic outliers that should be removed. The experimental results support the need for such QC.

Contour-VAE without QC could generate upcall-like contours, but its improvement in detector performance was limited. In contrast, VAE-QC α = 0.90 suppressed synthetic outliers using a more conservative threshold, but its detector performance was lower than that of DDPM. This indicates that overly strict QC can remove plausible variations useful for detector learning. VAE-QC α = 0.97 reduced outlier tails while preserving a wider range of real-like positive variation, and achieved the highest detector AUC. Thus, the proposed QC functions not as simple filtering, but as an acceptance mechanism for constructing a synthetic positive set suitable for detector training.

The DDPM results also support this interpretation. DDPM outperformed Original, indicating that a certain level of synthetic variation can help detector learning. However, DDPM-generated samples included some high-score tails in the feature and QC-score distributions, and DDPM does not directly control contour morphology or perform sample-level QC. In addition, because DDPM performs spectrogram-level generation, it can learn not only upcall patterns but also the background texture and noise statistics of the generator-side data.

VAE-QC also showed practical advantages in terms of computational cost. DDPM required approximately 80 s of generation time per sample, whereas Contour-VAE required approximately 2.01 s of end-to-end construction time per sample. This suggests that improving detector performance does not necessarily require a complex spectrogram-level generator; when the core morphology of the target signal is appropriately modeled, and the feature boundary is controlled through QC, a lightweight contour-based generator can also serve as an effective augmentation pipeline.

This study has several limitations. First, the experiments were conducted on a single public NARW benchmark dataset, and additional validation is required for external generalization across different recording sites, sensor types, sea areas, and seasonal conditions. Second, the proposed QC does not verify complete biological realism, and harmonic structure, source level, amplitude dynamics, propagation effects, and behavioral context are not included in the scope of this QC. Third, this study did not directly evaluate noise-type-specific robustness.

The proposed framework is primarily applicable to acoustic events with clear tonal contours, such as NARW upcalls. Future work should include cross-dataset validation using independent PAM datasets, human-in-the-loop contour extraction, noise-type-specific performance evaluation, and validation with different detector architectures. Nevertheless, this study shows that the core of generative augmentation lies not merely in generating synthetic samples, but in defining feature boundaries useful for detector learning and performing sample-level QC.

## 5. Conclusions

This study proposed a VAE-QC framework for NARW upcall detection. Instead of directly generating full raw waveforms or spectrograms, the proposed method learns manually extracted upcall frequency contours using a 1D-VAE and evaluates generated contours in a 10-dimensional acoustic morphology feature space. It also constructs a QC procedure that probabilistically accepts borderline samples around the hard threshold using real-contour score percentiles and same-call manual re-extraction variability.

Experiments using the Kaggle NARW dataset showed that VAE-QC α = 0.97 achieved the highest detector performance among Original, DDPM, Contour-VAE without QC, and VAE-QC α = 0.90. Feature distribution and QC-score analyses also showed that VAE-QC reduced the morphology-outlier tails observed in unfiltered generation and DDPM, and constructed a synthetic set more consistent with the real upcall contour distribution.

These results show that the key factor in generative augmentation is not simply generating many synthetic samples, but performing QC to distinguish positive variations useful for detector training from synthetic outliers that should be removed. Therefore, by combining contour-level representation and uncertainty-aware QC, this study demonstrates the practical potential of detector-oriented generative augmentation for NARW upcall detection.

## Figures and Tables

**Table 1:** Ten-dimensional acoustic morphology features extracted from each upcall contour.

Feature Symbol	Feature Meaning
$p_{1}$	Linear-fit slope
$p_{2}$	Linear-fit intercept
$N_{pk}$	Peak count
$f_{St}$	Start frequency
$f_{end}$	End frequency
$g_{mean}$	Mean slope
$g_{max}$	Maximum slope
$t_{Dur}$	Time duration
$f_{min}$	Minimum frequency
$f_{max}$	Maximum frequency

**Table 2:** Mean variability of the ten features.

Feature	Mean Variation
Linear-fit slope (${∆p}_{1}$)	109.5
Linear-fit intercept ($∆p_{2}$)	42.5
Peak count ($∆N_{pk}$)	2
Start frequency ($∆f_{St}$)	54.6
End frequency ($∆f_{end}$)	46.8
Mean slope ($∆g_{mean}$)	114.1
Maximum slope ($∆g_{max}$)	290.6
Time duration ($∆t_{Dur}$)	0.221
Minimum frequency ($∆f_{min}$)	54.6
Maximum frequency (${∆f}_{max}$)	46.8

**Table 3:** QC results under the 97% hard threshold setting ($\tau_{\alpha =0.97}^{data}$ = 4.1).

$Q_{\alpha }\left(s\left(f_{i}^{real}\right)\right)$	$s\left({\hat{f}}_{i}\right)$	$d_{i}$	$P_{acc}$
97%	$0\;<\;s\left({\hat{f}}_{i}\right)\leq$ 4.1	$d_{i}\leq$ 0	100%
98%	$4.1\;<\;s\left({\hat{f}}_{i}\right)\leq$ 10.94	$0\;<\;d_{i}\leq$ 6.84	92%
99%	$10.94\;<\;s\left({\hat{f}}_{i}\right)\leq$ 19.79	$6.84\;<\;d_{i}\leq$ 15.69	7%
100%	$19.79\;<\;s\left({\hat{f}}_{i}\right)\leq$ 46.84	$15.69\;<\;d_{i}\leq$ 42.74	0%

**Table 4:** QC results under the 90% hard threshold setting ($\tau_{\alpha =0.90}^{data}$ = 3.1).

$Q_{\alpha }\left(s\left(f_{i}^{real}\right)\right)$	$s\left({\hat{f}}_{i}\right)$	$d_{i}$	$P_{acc}$
90%	$0\;<\;s\left({\hat{f}}_{i}\right)\leq$ 3.1	$d_{i}\leq$ 0	100%
97%	$3.1\;<\;s\left({\hat{f}}_{i}\right)\leq$ 4.1	$0\;<\;d_{i}\leq$ 1	29%
98%	$4.1\;<\;s\left({\hat{f}}_{i}\right)\leq$ 10.94	$1\;<\;d_{i}\leq$ 7.84	66%
99%	$10.94\;<\;s\left({\hat{f}}_{i}\right)\leq$ 19.79	$7.84\;<\;d_{i}\leq$ 15.78	4%
		$15.78\;<\;d_{i}\leq$ 16.69	0%
100%	$19.79\;<\;s\left({\hat{f}}_{i}\right)\leq$ 46.84	$16.69\;<\;d_{i}\leq$ 43.74	0%

**Table 5:** Number of samples used for generator training.

Data Set		Upcall Sample
VAE-QC:	1D-VAE training	3000
	QC	514
1D-VAE training		3514
DDPM		3514

**Table 6:** Data partition for detector training protocol.

Data Set	Negative	Upcall	Usage
Full	22,973	7027	Baseline
Subset 1	11,486	3514	Generator and detector training
Subset 2	11,487	3513	Detector performance evaluation

**Table 7:** Detector training conditions for augmentation comparison.

Data Set	Negative	Upcall	
		Augmentation	Real
w.o. Augmentation	11,486	0	3514
VAE-QC α = 0.97		300	
VAE-QC α = 0.90		300	
VAE w.o. QC		300	
DDPM		300	

**Table 8:** Detection performance across augmentation conditions.

Method	AUC	Std (AUC)
w.o. Augmentation	0.802	0.004
VAE-QC α = 0.97	0.901	0.016
VAE-QC α = 0.90	0.824	0.008
VAE w.o. QC	0.810	0.010
DDPM	0.845	0.011

**Table 9:** Computational cost of DDPM and contour-based VAE-QC synthetic sample generation.

Method	Training Time	Per-Sample Generation Time	Additional Processing	Total Per-Sample Time
DDPM	~5 days	80.75 s	No	80.75 s
VAE-QC	~2 days	0.31 s (contour generation only)	~1.7 s (waveform reconstruction and spectrogram conversion)	2.01 s

## Data Availability

The Kaggle NARW dataset used in this study is publicly available at [21]. The detector is available at [24]. The manually extracted contours generated in this study can be accessed at https://zenodo.org/records/21205341 (20 July 2026). Other data are available from the corresponding author upon reasonable request, subject to institutional approval.

## Appendix A

To perform QC on the morphology of generated contours, this study extracted a 10-dimensional feature vector from each frequency contour. This appendix describes the feature extraction procedure in detail. After the sample length of the extracted contour is normalized to L, smoothing is applied. The resulting contour is defined as in Equation (A1).

(A1) $$\left\{\begin{array}{c} f=[f_{1},\ldots ,f_{L}]\\ t=[t_{1},\ldots ,t_{L}] \end{array}\right..$$

In Equation (A1), f denotes the contour frequency value, and t denotes the time value corresponding to f. In this paper, L was set to 128. All features are then computed using the contour defined in Equation (A1). First, a first-order linear model is fitted to the contour. In this paper, the first-order linear model was fitted using least-squares fitting. The resulting linear model can be expressed as Equation (A2).

(A2) $$f=p_{1}t+p_{2}.$$

Here, $p_{1}$ is the overall frequency trend of the contour, i.e., the linear-fit slope of the upcall, and $p_{2}$ is the linear-fit intercept, representing the baseline level of the linear trend. Next, to represent the local curvature structure of the contour, the numbers of local maxima and local minima are computed. If the numbers of local maxima and local minima are denoted by $N_{max}$ and $N_{min}$, respectively, the Peak count representing the local turning structure of the contour is obtained as follows.

(A3) $$N_{pk}=N_{max}+N_{min}.$$

To represent the temporal rate of change of the contour, the gradient of the smoothed contour is computed with respect to the time axis, and the mean and maximum values of the gradient sequence are used as features. The mean value of the gradient sequence is obtained as in Equation (A4).

(A4) $$g_{mean}=\frac{1}{L-1}\sum_{n=1}^{L-1}\frac{f_{n+1}-f_{1}}{t_{n+1}-t_{1}}.$$

Similarly, the maximum value of the gradient sequence is obtained as in Equation (A5).

(A5) $$g_{max}=\underset{1\leq n\leq L-1}{\max}\frac{f_{n+1}-f_{1}}{t_{n+1}-t_{1}}$$

Here, $g_{mean}$ denotes the mean slope of the contour, and $g_{max}$ denotes the maximum slope in the interval with the steepest frequency increase. In addition, contour duration, start and end frequencies, and minimum and maximum frequencies are used as features, as shown in Equation (A6).

(A6) $$\left\{\begin{array}{c} \begin{array}{c} t_{Dur}=t_{L}\\ \begin{array}{c} f_{St}=f_{1}\\ f_{end}=f_{L} \end{array} \end{array}\\ \begin{array}{c} f_{max}=max(f)\\ f_{min}=min(f) \end{array} \end{array}\right..$$

The features defined above are combined to form the final 10-dimensional contour feature vector.
